# Virulence Associated Gene 8 of *Bordetella pertussis* Enhances Contact System Activity by Inhibiting the Regulatory Function of Complement Regulator C1 Inhibitor

**DOI:** 10.3389/fimmu.2018.01172

**Published:** 2018-06-04

**Authors:** Elise S. Hovingh, Steven de Maat, Alexandra P. M. Cloherty, Steven Johnson, Elena Pinelli, Coen Maas, Ilse Jongerius

**Affiliations:** ^1^Department of Medical Microbiology, University Medical Centre Utrecht, Utrecht University, Utrecht, Netherlands; ^2^Centre for Infectious Disease Control, National Institute for Public Health and the Environment (RIVM), Bilthoven, Netherlands; ^3^Department of Clinical Chemistry and Haematology, University Medical Centre Utrecht, Utrecht University, Utrecht, Netherlands; ^4^Sir William Dunn School of Pathology, University of Oxford, Oxford, United Kingdom

**Keywords:** whooping cough, contact system, virulence associated gene 8, *Bordetella pertussis*, C1 inhibitor

## Abstract

*Bordetella pertussis* is a Gram-negative bacterium and the causative agent of whooping cough. Whooping cough is currently re-emerging worldwide and, therefore, still poses a continuous global health threat. *B. pertussis* expresses several virulence factors that play a role in evading the human immune response. One of these virulence factors is virulence associated gene 8 (Vag8). Vag8 is a complement evasion molecule that mediates its effects by binding to the complement regulator C1 inhibitor (C1-INH). This regulatory protein is a fluid phase serine protease that controls proenzyme activation and enzyme activity of not only the complement system but also the contact system. Activation of the contact system results in the generation of bradykinin, a pro-inflammatory peptide. Here, the activation of the contact system by *B. pertussis* was explored. We demonstrate that recombinant as well as endogenous Vag8 enhanced contact system activity by binding C1-INH and attenuating its inhibitory function. Moreover, we show that *B. pertussis* itself is able to activate the contact system. This activation was dependent on Vag8 production as a Vag8 knockout *B. pertussis* strain was unable to activate the contact system. These findings show a previously overlooked interaction between the contact system and the respiratory pathogen *B. pertussis*. Activation of the contact system by *B. pertussis* may contribute to its pathogenicity and virulence.

## Introduction

*Bordetella pertussis* is the causative agent of whooping cough, also known as pertussis, a contagious disease of the respiratory tract that is re-emerging worldwide despite high vaccination coverage. To date, pertussis is still ranked in the top 10 most deadly childhood diseases posing a serious health problem (1). The acellular pertussis vaccine (ACV), used in many industrialized countries, protects against disease for up to 7 years while natural infection confers protection for up to 20 years (2, 3). Alarmingly, the ACV does not prevent transmission of the pathogen (4). For this reason, it is widely accepted that an improved pertussis vaccine is needed (5). In order to improve the pertussis vaccine, it is of great importance to better understand the interactions between the respiratory pathogen *B. pertussis* and the immune system.

The contact system is a key player in innate immunity and is part of the coagulation system (6, 7). The contact system consists of the two proenzymes factor XII (FXII) and plasma prekallikrein and the cofactor high-molecular weight kininogen (HK). *In vitro*, the contact system is activated when FXII binds to a negatively charged surface and is autocleaved forming FXIIa that is further processed to βFXIIa (8). Lessons from human pathology imply that analogous processes may take place on the surface of vascular endothelial cells (9) or platelets (10). FXIIa cleaves plasma prekallikrein forming active plasma kallikrein (PK). Activation of this protease subsequently mediates the cleavage of HK and formation of the pro-inflammatory peptide bradykinin (BK) (11). BK release triggers endothelial permeability resulting in vasodilation and infiltration of leukocytes (7). Activation and activity of the contact system is regulated by the 105 kDa complement regulator C1 inhibitor (C1-INH) (12), which inhibits the activity of β-FXIIa, α-FXIIa, and PK, by forming covalent complexes with its target proteases (13). C1-INH consists of a C-terminal protease inhibiting serpin domain and an N-terminal domain that is predicted to be heavily O- and N-linked glycosylated. Besides being involved in contact system regulation, C1-INH is also the main inhibitor of the classical and lectin pathways of the complement system where it inactivates the respective proteases necessary for activation of the complement cascade (14). Interestingly, the interplay between *B. pertussis* and the contact system remains unexplored even though *Escherichia coli* and *Salmonella* (15), *Streptococcus pyogenes, Bacillus stearothermophilus, Bacillus subtilis, Porphyromonas gingivalis, Pseudomonas aeruginosa, Serratia maracescens*, as well as several Vibrio species (16) have been shown to activate this system.

*B. pertussis* produces multiple virulence factors involved in immune evasion (17). It was recently shown that virulence associated gene 8 (Vag8) of *B. pertussis* binds to C1-INH (18, 19). Vag8 is a 95 kDa autotransporter. Autotransporters are typically processed into a channel and a passenger domain (20). The passenger domain will pass through the channel and can either remain attached to the bacterial membrane or be secreted into the bacterial surrounding (21). Autotransporter proteins, including Vag8, are also present on the surface of outer membrane vesicles (OMVs) that are secreted by Gram-negative bacteria (18, 22, 23). We have recently shown that secreted Vag8 binding to C1-INH away from the bacterial surface leads to complement evasion. This binding result in consumption of complement components C2 and C4 *via* uncontrolled cleavage by the proteases C1r, C1s, and MASP-2, away from the bacterial surface (18).

Since C1-INH controls both the complement and the contact system, we here investigated whether Vag8 influences contact system activity. We demonstrate that both recombinant and endogenously secreted Vag8 enhanced contact system activity by attenuation of the inhibitory function of C1-INH. Moreover, we show that *B. pertussis* effectively activated the contact system by producing Vag8.

## Materials and Methods

### Bacterial Strains and Growth Conditions

*B. pertussis* wild type B1917 strain (isolated in 2000), the isogenic Vag8 knockout strain B1917ΔVag8 (18), the B0442 strain producing a mutated lipooligosaccharide (LOS) that was isolated in 1954 (24) and the pertactin-deficient B4418 and B4374 strains as well as the pertactin-producing B4430 and B4393 strains isolated in 2016 were grown at 35°C, 5% CO_2_ on Bordet Gengou plates containing glycerol and 15% defibrinated sheep blood (BD Biosciences, Franklin Lakes, NJ, USA). After 3–5 days of culture, the bacteria were collected in buffer containing 50 mM HEPES, 2 mM CaCl_2_, 50 µM ZnCl_2_, 0.02% NaN_3_, and 0.05% Tween-20 (pH 7.35) further referred to as buffer A, the optical density was measured at 600 nm and bacteria were washed in buffer A. OMVs of both strains were prepared by ultracentrifugation as described previously (18, 25, 26).

### Recombinant Production of Histidine-Tagged (his-tag) Vag8 and the Negative Control *Bordetella* Resistance to Killing A (BrkA) Passenger Domain

Recombinant his-tag passenger domain of Vag8 was produced as previously described (18). BrkA was cloned using primers 5′-ATATGGATCCCAGGAAGGAGAGTTCGAC-3′ and 5′-ATATGCGGCCGCCTACTGCAAGCTCCAGACATG-3′ (restriction sites underlined) and ligated into a modified pRSET-B vector containing a non-cleavable six residue his-tag (MHHHHHHGS) at the N-terminus of the protein as described previously (18, 27). BrkA was expressed and purified as Vag8 (18).

### Surface Plasmon Resonance (SPR)

SPR was performed using a Biacore T200 (GE Healthcare, Little Chalfont, UK). Recombinant passenger domain of Vag8 was dissolved in 50 mM sodium acetate pH 5.0 and immobilized using primary amine coupling onto a CM5 sensor chip (GE Healthcare). All binding experiments were performed at 25°C in 10 mM HEPES pH 7.4, 150 mM NaCl, 3 mM EDTA, 0.005% (v/v) surfactant P20. Increasing concentrations (2.5–160 nM) of either full length C1-INH (C1-INH_FL_, Complement Technology) or C1-INH containing only the serpin domain (C1-INH_NT98_) (28) were injected over the flow channels at 30 µL/min. Dissociation was allowed for 300 s followed by surface regeneration with 10 mM glycine pH 2.5. BIAevaluation software (GE Healthcare) was used to analyze the data.

### Size Exclusion Chromatography With Multi-Angle Light Scattering Analysis (SEC-MALS)

100 µL of protein samples were injected onto an S200 increase 10/300 column (GE Healthcare) equilibrated in 50 mM Tris pH 7.5, 150 mM NaCl and eluted with a flow rate of 0.4 mL/min. Light scattering and refractive index changes were measured using a Dawn Heleos-II light scattering detector and an Optilab-TrEX refractive index monitor respectively. Analysis was carried out using ASTRA 6.1.1.175.3.4.14 software assuming a dn/dc value of 0.186 mL/g.

### Ethics

The study was conducted using blood donation from ±50 healthy adults for plasma collection and according to the principles expressed in the Declaration of Helsinki. Written informed consent was obtained from all blood donors before collection and samples were used anonymously. Approval was obtained from the medical ethics committee of the University Medical Centre Utrecht.

### Plasma

Blood was collected in blood tubes containing sodium citrate (Vacuette tube, Greiner Bio-one, Kremsmünster, Austria) from ±50 healthy volunteers after written informed consent. Following collection, samples were centrifuged twice at 2,000 *g* for 10 min and plasma of all donors was pooled. The pooled plasma was stored in aliquots at −80°C until use.

### Activation of the Contact System: Chromogenic Substrate Assay

To determine whether Vag8 could interfere with the inhibitory function of C1-INH on βFXIIa and PK, we made use of the chromogenic substrate H-D-Pro-Phe-Arg-pNA (L-2120, Sigma (Merck), Darmstadt, Germany) that can be cleaved by both proteases (29). All experiments were performed in 96-well PVC flat-bottom microplate (Corning GmbH, Wiesbaden, Germany). Activation of the contact system in the presence of Vag8 and BrkA was first analyzed in a purified system. βFXIIa (33.3 nM corresponding to 1 µg/mL, Enzyme Research Laboratories, South Bend, Ind, USA) or PK (5.81 nM corresponding to 0.5 µg/mL, Enzyme Research Laboratories) was pre-incubated with or without serum-derived C1-INH (95.2 nM corresponding to 10 µg/mL, Complement Technologies, Tyler, TX, USA) that was pre-incubated for 10 min at 37°C with, Vag8, BrkA, buffer A (for βFXIIa), or phosphate buffered saline (PBS) (for PK). Activity was measured following the addition of 0.5 mM L-2120 (Bachem, Bubendorf, Switzerland) (29). Activation of the contact system in a more complex system was studied in citrated human plasma. For these experiments, 60% plasma was activated with βFXIIa (16.7 nM corresponding to 0.5 µg/mL) in the presence of PBS (buffer control), Vag8, BrkA (concentrations indicated in the figures), or OMVs obtained from the *B. pertussis* wild type strain B1917 or knockout strain B1917ΔVag8 (60 µg/mL) with 10 min pre-incubation at 37°C before addition of the chromogenic substrate L-2120. For maximum contact system activity, referred to as control, βFXIIa was added to the plasma at the same time as the addition of L-2120 ensuring that C1-INH did not have the chance to inhibit the contact proteases. The substrate conversion, referred to as kallikrein-like activity, was measured in a kinetic fashion up until 30 min (Figure S1 in Supplementary Material) and reported at the 10 min time point. Substrate conversion was measured with a microplate reader at 405 nm at 37°C over time (VersaMax microplate reader, Molecular Devices, Sunnyvale, CA, USA).

Activation of the contact system by *B. pertussis* was assessed by incubating *B. pertussis* wild type strain B1917 or knockout strain B1917ΔVag8 (3 × 10^7^ CFU each) with 60% plasma or buffer A. Plasma incubated with βFXIIa (16.7 nM corresponding to 0.5 µg/mL, Enzyme Research Laboratories) or only buffer A was taken along as positive and negative controls, respectively. These experiments were performed with 5 min pre-incubation at 37°C before addition of the substrate L-2120. Substrate conversion was measured at 37°C every 30 s with a microplate reader at 405 nm over time (PowerWave XS Microplate Spectrophotometer, BioTek, Winooski, VT, USA).

### Cleavage of HK: Immunoblotting

To determine cleavage of HK in plasma in the presence of Vag8, βFXIIa (16.7 nM corresponding to 0.5 µg/mL) was added to 90% plasma and incubated with either PBS or Vag8 (1,017 nM corresponding to 60 µg/mL) for 10 min at 37°C. Samples were diluted 40 times in reducing sample buffer (15.5% glycerol, 96.8 mM Tris–HCl, 3.1% SDS, 0.003% bromophenol blue, and 25 mM DTT), incubated for 10 min at 100°C and separated on a 10% SDS-PAGE gel. Proteins were blotted onto PVDF membranes. Membranes were blocked with 4% skimmed milk in PBS containing 0.05% Tween (PBS-T) and washed with PBS-T three times for 10 min at 37°C between each incubation step. The immunoblot was subsequently incubated with a primary goat anti-human HK antibody (3 µg/mL final concentration, Affinity Biologicals, Ancaster, ON, Canada) overnight at 4°C and a secondary donkey-anti-goat-HRP antibody (0.5 µg/mL, Southern Biotech, Birmingham, AL, USA) mainly reacting with the HK light chain (30) for 2 h at 37°C. All antibodies were diluted in 1% skimmed milk in PBS-T. For detection, the Pierce ECL Western Blotting Substrate (Thermofisher Scientific, Waltham, MA, USA) was used and visualized using the ImageQuant (GE Life Sciences, Chicago, IL, USA).

For assessment of HK cleavage upon incubation of plasma with *B. pertussis*, several bacterial strains (2 × 10^9^ CFU) were incubated with 50% plasma either alone or in the presence of the contact protease inhibitors aprotinin (31) (100 units/mL, Sigma), which inhibits PK and d-phenylalanyl-prolyl-arginyl chloromethyl ketone (32) (PPACK; 200 µM, Hematologic Technologies, Essex Junction, VT, USA), a multi-target serine protease inhibitor which restricts auto activation and self-digestion of FXIIa, and sampled after 30 min at 37°C shaking at 300 rpm. Plasma samples incubated with βFXIIa (16.7 nM corresponding to 0.5 µg/mL) or only buffer A were taken along as positive and negative controls, respectively. Samples were analyzed as described above.

### Statistical Analyses

Statistical analyses were performed using GraphPad Prism 6.02 and the differences between groups were analyzed for significance using the two-tailed Student’s *t*-test. A *p*-value of ≤0.05 was considered statistically significant.

## Results

### Vag8 Attenuates the Role of C1-INH as Inhibitor of βFXIIa and PK

**Figure 1:**
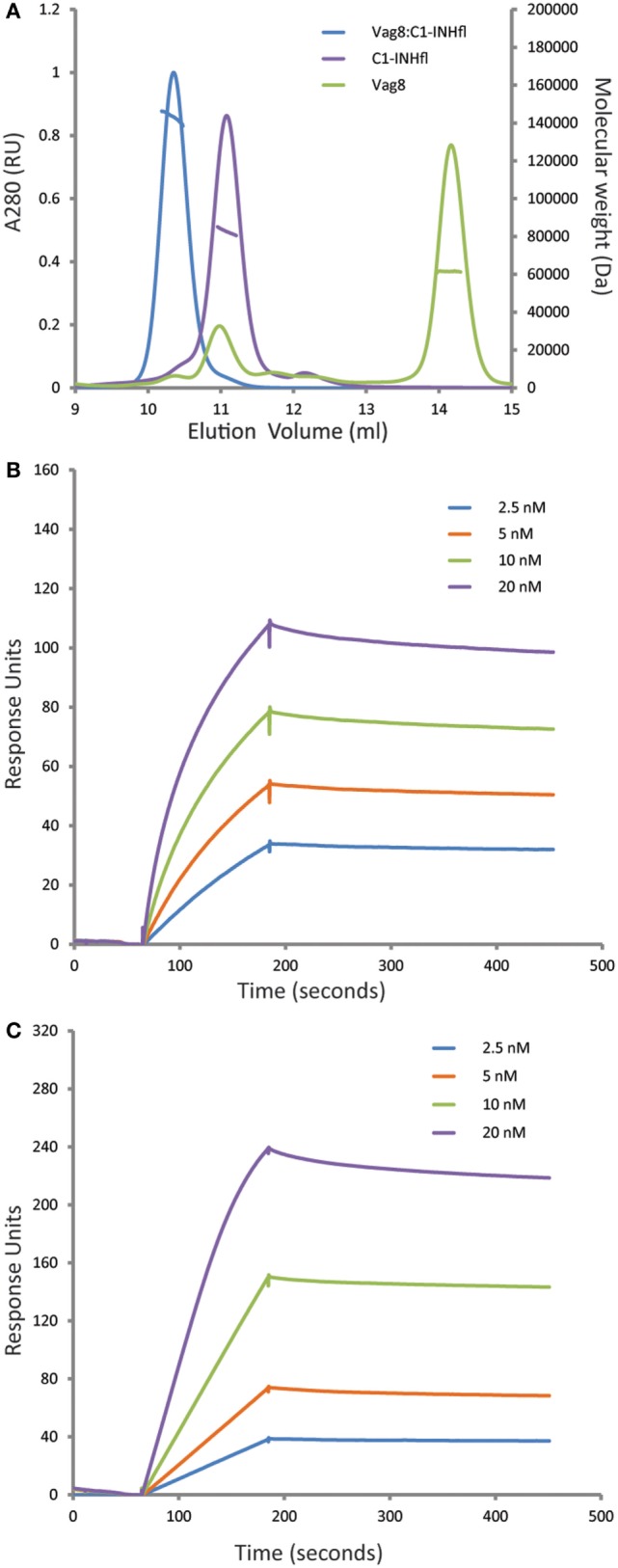
Virulence associated gene 8 (Vag8) and complement regulator C1 inhibitor (C1-INH) interact to form a tight 1:1 complex. **(A)** Size exclusion chromatography with multi-angle light scattering analysis (SEC-MALS) of Vag8 (green), C1-INH (purple), and Vag8_C1-INH complex (blue). The masses calculated from the scattering are shown as lines across the peaks. Protein conjugate analysis demonstrates that the mass of complex (146 kDa) corresponds to 118 kDa of protein and 28 kDa of sugar. This is consistent with a 1:1 complex of Vag8 (60 kDa) and glycosylated C1-INH (58 kDa protein/28 kDa sugar). **(B,C)** SPR analyses of the binding between Vag8 on the chip surface and increasing concentrations of C1-INH_FL_ **(B)** and C1-INH_NT98_ **(C)** in solution.

It was recently shown that Vag8 binds to C1-INH using both ELISA and gel filtration chromatography (18, 19). In order to further characterize this interaction, we performed the combination of SEC-MALS on the individual proteins and the complex (Figure 1A). Both Vag8 and C1-INH behaved as monomers on the column. Addition of Vag8 to C1-INH shifted the elution position and calculation of the mass across the peak revealed that a 1:1 complex had been formed. We next performed SPR analysis to attempt to assess the affinity of the interaction (Figures 1B,C). Flowing increasing concentrations of C1-INH_FL_ over Vag8 on the surface demonstrated clear binding. The almost non-existent off-rate of the interaction, combined with a slow enough on-rate that precluded reaching equilibrium, meant that we were unable to robustly calculate the K_D_ of the interaction. However, attempts to fit the kinetics using a variety of binding models always produced K_D_ values of 1 nM or lower, consistent with a tight interaction. In order to confirm that the interaction between Vag8 and C1-INH involved the serpin domain, we repeated the SPR with C1-INH lacking the N-terminal O-linked glycosylation domain. This construct interacted with Vag8 with very similar kinetics to the full length protein (Figure 1C).

**Figure 2:**
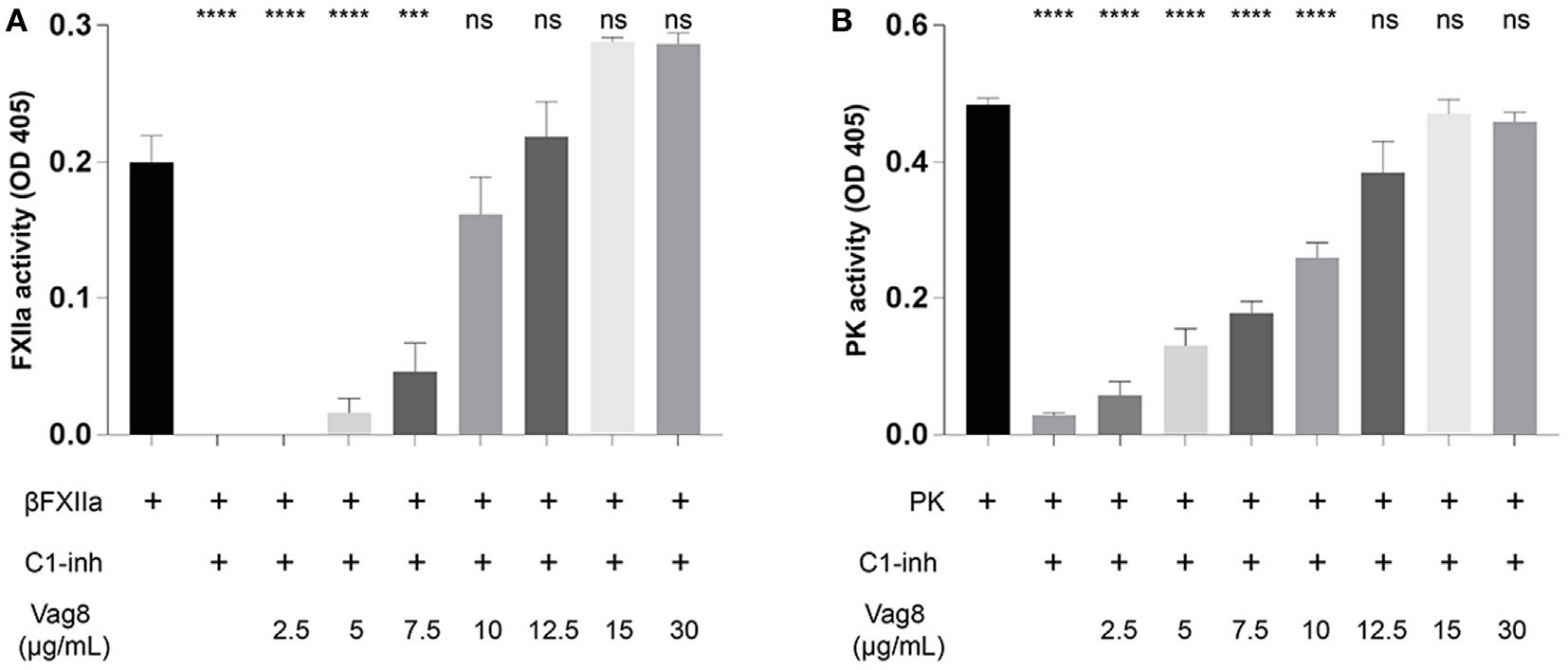
Virulence associated gene 8 (Vag8) interferes with the inhibition of βFXIIa and plasma kallikrein (PK) by complement regulator C1 inhibitor (C1-INH). **(A)** Vag8 (2.5, 5, 7.5, 10, 12.5, 15, and 30 µg/mL) dose-dependently enhances the activity of 1 µg/mL βFXIIa in the presence of 10 µg/mL C1-INH reaching similar activity as βFXIIa alone upon using 10 µg/mL of Vag8. **(B)** Vag8 (2.5, 5, 7.5, 10, 12.5, 15, and 30 µg/mL) dose-dependently enhances the activity of 0.5 µg/mL PK in the presence of 10 µg/mL C1-INH reaching similar activity as PK alone upon using 12.5 µg/mL of Vag8. Data represent the mean ± SEM of three separate experiments. ***p ≤ 0.001, ****p ≤ 0.0001, ns = non-significant compared to the black bar.

We have demonstrated that the binding of secreted Vag8 to C1-INH results in attenuation of the inhibitory effect of C1-INH leading to the cleavage of essential complement proteins away from the bacterial surface (18). Since C1-INH is also one of the main inhibitors of the contact system, we hypothesized that the interaction between C1-INH and Vag8 would have a similar effect on the activation of the contact system. To investigate the effect of Vag8 on the contact system, we first studied this in a purified system. Addition of Vag8 to purified βFXIIa and C1-INH resulted in a dose-dependent enhanced conversion of the chromogenic substrate. Addition of 10 µg/mL Vag8 (169.5 nM) is sufficient to completely neutralize C1-INH activity as comparable levels of activation as that of βFXIIa alone were reached (Figure 2A). This dose-dependent attenuation of inhibition by C1-INH was also observed upon the addition of Vag8 to a purified system containing PK and C1-INH (Figure 2B). A concentration of 12.5 µg/mL Vag8 (203.4 nM) was sufficient to completely block the inhibitory capacity of C1-INH on PK activation. As a negative control we used BrkA. BrkA is another autotransporter protein of *B. pertussis* involved in complement evasion. BkrA has a similar structure and size as Vag8 and was produced in a similar way as Vag8 (33, 34). The inhibitory properties of C1-INH on βFXIIa and PK were not disturbed by the addition of BrkA at equimolar concentrations (Figure S2 in Supplementary Material). Taken together, Vag8 dose dependently attenuates the inhibitory effect of C1-INH on βFXIIa and PK in a purified system.

### Vag8 Enhances Contact System Activity in Plasma

**Figure 3:**
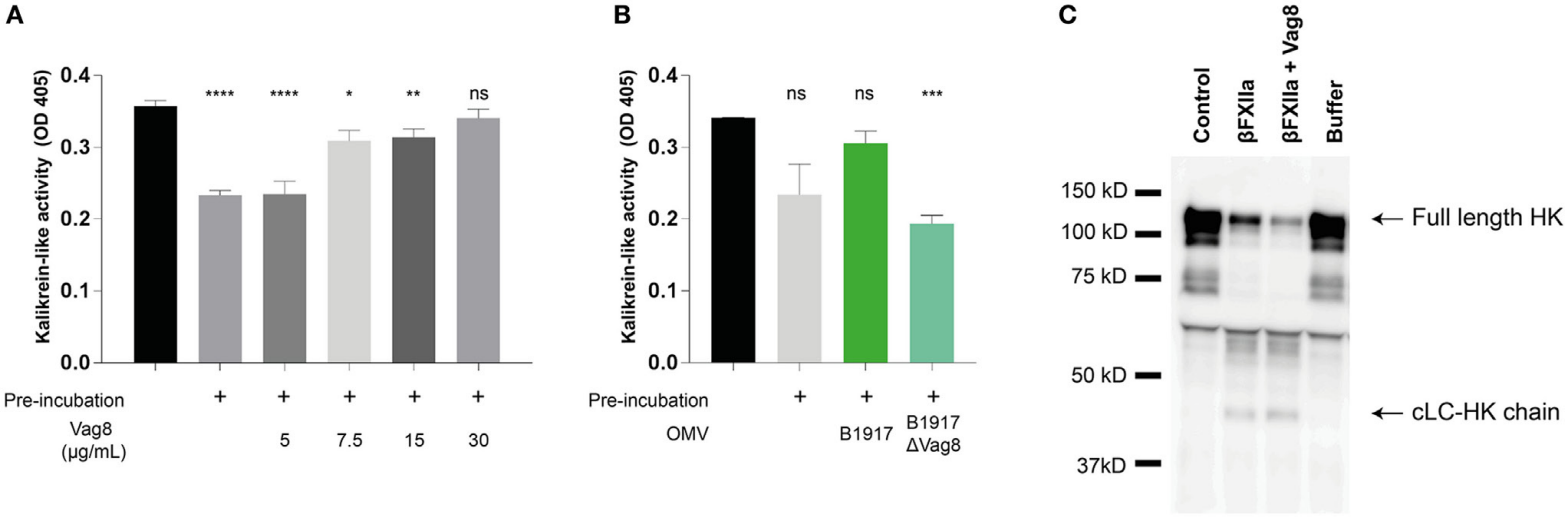
Virulence associated gene 8 (Vag8) induces enhanced activation of the contact system in plasma. **(A)** 60% plasma was incubated with Vag8 (5, 7.5, 15, and 30 µg/mL) following 10 min pre-incubation with 0.5 µg/mL βFXIIa. Vag8 dose-dependently enhances the induced kallikrein-like activity reaching similar levels upon using 7.5 µg/mL of Vag8 as the maximum kallikrein-like activity observed by βFXIIa alone without pre-incubation. **(B)** Moreover, the kallikrein-like activity in 60% plasma was also enhanced in the presence of endogenous Vag8 on outer membrane vesicles (OMVs) (60 µg/mL) derived from the wild type *Bordetella pertussis* strain B1917 but not when using OMVs of the knockout strain B1917ΔVag8. **(C)** Enhanced contact system activation was also shown by immunoblot. 90% plasma was incubated with 0.5 µg/mL βFXIIa either in the presence of 60 µg/mL Vag8 or buffer for 10 min and analyzed using anti-high-molecular-weight-kininogen (HK). The presence of Vag8 results in increased cleavage of the ~120 kDa full length HK compared to βFXIIa alone and the detection of the ~46 kDa cLC-HK chain (note the second and third lane). Plasma alone at *t* = 0 (control) and without βFXIIa at *t* = 10 (buffer) showed no cleavage of HK. Data shown in panel **(A)** represent the mean ± SEM of three separate experiments, while panels **B** and **C** are representative of three separate experiments. *p ≤ 0.05, **p ≤ 0.01, ***p ≤ 0.001, ****p ≤ 0.0001, ns, non-significant compared to the black bar.

Since we have shown that Vag8 can interfere with the regulatory activity of C1-INH on βFXIIa and PK in a purified system, we next assessed the effect of Vag8 in a more physiological setting. To this end, we incubated plasma either with buffer (pre-incubated plasma) or increasing concentrations of Vag8 in the presence of the contact system activator βFXIIa for 10 min before addition of the chromogenic substrate. This pre-incubation step is needed for C1-INH to inhibit the contact proteases βFXIIa and PK. Moreover, the chromogenic substrate was added immediately following βFXIIa addition (control plasma) indicating the maximum kallikrein-like activity. Figure 3A shows that Vag8 dose-dependently attenuates the inhibitory function of C1-INH thus enhancing the activation of the contact system, referred to as kallikrein-like activity. Comparable levels as control plasma are reached upon using 7.5 µg/mL of Vag8 (127.1 nM), with no significant difference between the control plasma and addition of 30 µg/mL of Vag8 (508.5 nM). The negative control, BrkA, did not show any effect on contact system activity (data not shown). To determine whether endogenously secreted Vag8 is also capable of mediating this effect, OMVs derived from *B. pertussis* wild type strain B1917 or the knockout strain B1917ΔVag8 (18) were incubated with plasma and βFXIIa. We show that the OMVs derived from *B. pertussis* wild type strain B1917 were capable of attenuating the inhibitory function of C1-INH resulting in enhanced contact system activity to levels observed in control plasma conditions, whereas the OMVs obtained from the knockout strain B1917ΔVag8 were not (Figure 3B).

As previously mentioned, activation of the contact system ultimately results in the cleavage of HK and the subsequent release of BK. Also clinically, cleavage of HK is related to BK production (30). To investigate the effect of recombinant Vag8 binding to C1-INH on HK cleavage in plasma, βFXIIa was incubated with plasma in the presence or absence of Vag8 and was assessed by immunoblotting to visualize HK cleavage. Figure 3C shows increased HK cleavage as indicated by the decreased intensity of the ~120 kDa full length HK band (30) in the presence of Vag8 compared to incubation with βFXIIa alone as well as by the detection of the ~46 kDa cLC-HK chain. This is the cleaved L-chain which is often used as a marker of extensive contact system activation in plasma (35). In conclusion, we show enhanced contact system activity in the presence of recombinant and endogenous Vag8, which is most likely due to Vag8 binding to C1-INH and hence hampering the inhibitory properties of C1-INH on βFXIIa and PK.

### *B. pertussis* Activates the Contact System Predominantly Through Vag8 Production

**Figure 4:**
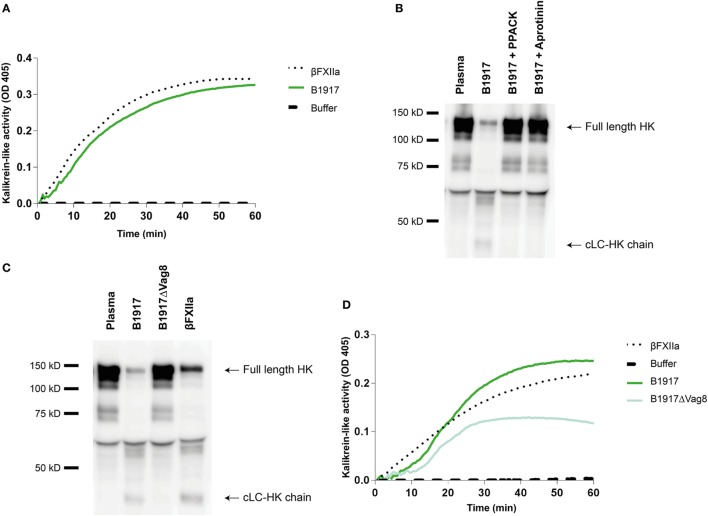
*Bordetella pertussis* activates the contact system mainly by Virulence associated gene 8 (Vag8) production. **(A)** *B. pertussis* wild type strain B1917 (3 × 10^7^ CFU) induces similar kallikrein-like activity in 60% plasma compared to addition of 0.5 µg/mL βFXIIa. **(B)** Contact system activation by *B. pertussis* wild type strain B1917 was also shown by immunoblot. 50% plasma was incubated for 60 min with *B. pertussis* wild type strain B1917 (2 × 10^9^ CFU) alone or in combination with the contact system inhibitors d-phenylalanyl-prolyl-arginyl chloromethyl ketone (PPACK) or aprotinin and analyzed using anti-high-molecular-weight-kininogen (HK). Incubation with *B. pertussis* wild type strain B1917 resulted in almost complete cleavage of the ~120 kDa full length HK as indicated by the appearance of the ~46 kDa cLC-HK chain. This feature was not observed in the presence of the inhibitors. **(C)** To determine whether Vag8 production was involved in contact system activation by this pathogen, 50% plasma was incubated either with *B. pertussis* wild type strain B1917 or with the knockout strain B1917ΔVag8 (2 × 10^9^ CFU) and analyzed using anti-HK. The *B. pertussis* knockout strain B1917ΔVag8 was unable to activate the contact system as no cleavage of the ~120 kDa full length HK was detected. **(D)** This was also shown by the decreased kallikrein-like activity in 60% plasma upon incubation with the knockout strain B1917ΔVag8 (3 × 10^7^ CFU) compared to the *B. pertussis* wild type strain B1917. Panels **A–D** are representative of three separate experiments.

**Figure 5:**
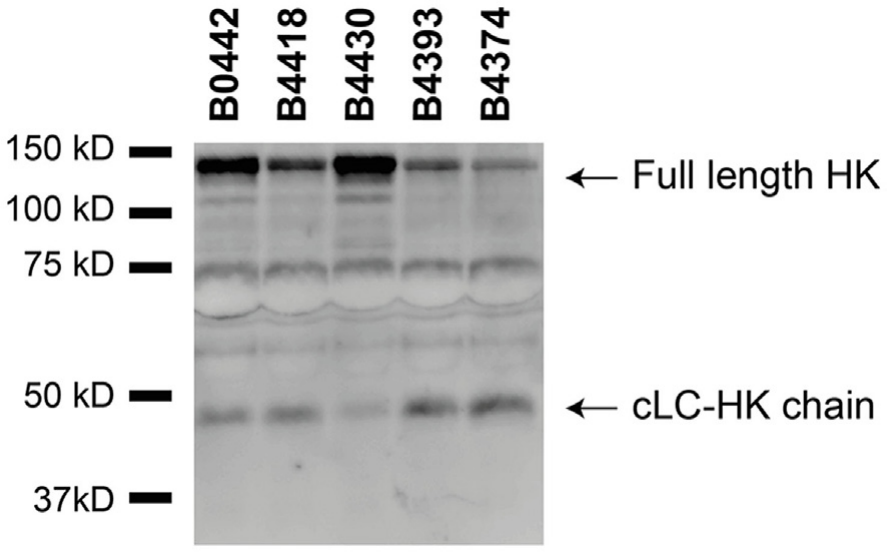
Various *Bordetella pertussis* strains activate the contact system. Contact system activation by *B. pertussis* strains B0442, B4418, B4430, B4393, and B4374 was shown by immunoblot. 50% plasma was incubated for 60 min with the *B. pertussis* strains (2 × 10^9^ CFU) and analyzed using anti-high-molecular-weight-kininogen (HK). Incubation with all the *B. pertussis* strains resulted in cleavage of the ~120 kDa full length HK as indicated by the appearance of the ~46 kDa cLC-HK chain.

Although several bacteria are known to activate the contact system (15, 36–42), the interaction between *B. pertussis* and the contact system has not been investigated. As shown above, Vag8 of *B. pertussis* hampers the inhibition of the contact proteases by binding C1-INH and hence enhances contact system activity (Figures 2 and 3). Next, we investigated whether *B. pertussis* itself can effectively activate the contact system. We show that *B. pertussis* wild type strain B1917 successfully activates the contact system in plasma as an increase in kallikrein-like activity was observed over time (Figure 4A). This activation was further examined by assessment of HK cleavage using immunoblot. Figure 4B shows a representative immunoblot in which degradation of the ~120 kDa full length HK and appearance of a ~46 kDa cLC-HK chain (30) was observed when *B. pertussis* strain B1917 was added to the plasma. To verify that the observed HK cleavage was the result of contact system activation, aprotinin (31) and PPACK (32) were added to the B1917 samples to prevent FXIIa and PK activity. Figure 4B shows a lack of HK cleavage upon addition of both these inhibitors to plasma incubated with *B. pertussis* wild type strain B1917 indicating that the HK cleavage observed in the presence of this bacterium can be attributed to the activation of the contact system. Next, we assessed whether Vag8 production was responsible for the activation of the contact system by *B. pertussis*. Figure 4C shows the lack of HK cleavage in the presence of the *B. pertussis* knockout strain B1917ΔVag8 by immunoblot. Even after 180 min of incubation, no activation of the contact system by the *B. pertussis* knockout strain B1917ΔVag8 was observed (data not shown). This is further supported by the decreased kallikrein-like activity shown upon incubating plasma with the *B. pertussis* knockout strain B1917ΔVag8 when compared to incubation with wild type strain B1917 (Figure 4D). Moreover, we show that contact system activation is not restricted to *B. pertussis* strain B1917. Incubation of plasma with the LOS-mutant B0442 as well as clinical strains either producing (B4430 and B4393) or not producing pertactin (B4418 and B4374) also shows contact system activation as indicated by the detection of the ~46 kDa cLC-HK chain (Figure 5). In summary, *B. pertussis* is capable of activating the contact system as demonstrated by the observed kallikrein-like activity as well as by the cleavage of HK detected by immunoblot. We show that this is mainly dependent on the production of the autotransporter Vag8, which will bind to C1-INH and thus attenuate its inhibitory function, as the *B. pertussis* knockout strain B1917ΔVag8 showed no HK cleavage and decreased kallikrein-like activity compared to its isogenic wild type strain B1917.

## Discussion

**Figure 6:**
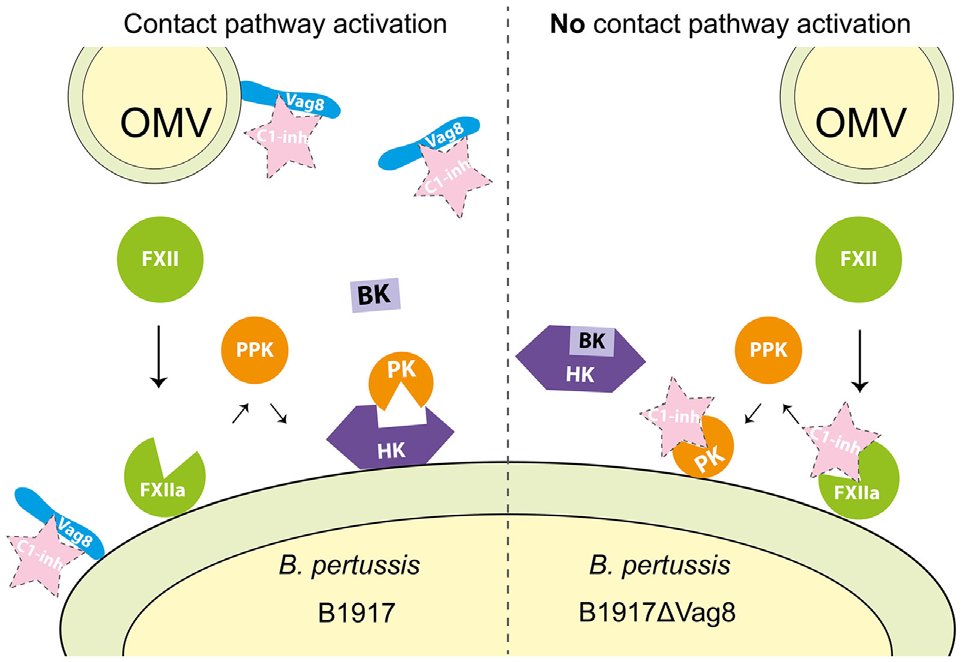
Proposed mechanism for Virulence associated gene 8 (Vag8) mediated activation of the contact system. Vag8, either on the bacterial surface as part of an outer membrane vesicles (OMVs) or as the secreted passenger, binds to complement regulator C1 inhibitor (C1-INH) (left panel). This results in the lack of inhibition of the contact system proteases FXIIa and plasma kallikrein (PK) by C1-INH. The lipooligosaccharide and polyphosphates present on the outer membrane of *Bordetella pertussis* are most likely responsible for FXII activation on the bacterial membrane as has been shown for other bacteria (15, 36–42). This activation, which cannot be inhibited by C1-INH as it is bound to Vag8, will result in the release of bradykinin (BK). In the absence of Vag8, C1-INH will inhibit FXIIa and PK when formed and high-molecular-weight-kininogen (HK) will remain intact away from the bacterial surface (right panel).

Recently, we unraveled the mechanism responsible for secreted Vag8-mediated complement evasion (18). We showed that binding of secreted Vag8 to C1-INH resulted in the release of the active proteases C1s, C1r, and MASP-2 since C1-INH could no longer bind and thus inhibit their proteolytic activity. The presence of active C1s, C1r, and MASP-2 proteases in the serum resulted in the degradation of the complement proteins C4 and C2 away from the bacterial surface (18). We suggest that *B. pertussis* uses this complement evasion strategy to aid in the prevention of opsonization and complement-mediated lysis. Vag8 is not only secreted but also present on the bacterial surface where it binds to C1-INH (19, 43). Binding of C1-INH to the bacterial surface could also result in the inhibition of complement activation which has been shown for other bacteria such as *Borrelia recurrentis* (44). The contact system is another innate immune component consisting of proteases (11). Here, we show that in addition to interacting with the complement system (18), Vag8 of *B. pertussis* induced enhanced activation of the contact system as demonstrated by increased contact system activity and HK cleavage. We propose that Vag8 mediates activation of the contact pathway by binding to C1-INH and attenuating its inhibitory function as we have previously shown for its effect on the complement system (18). C1-INH is the predominant fluid phase inactivator of FXIIa and PK. This serpin inactivates these proteases by irreversibly binding to them resulting in conformational changes that disrupt the active site of the target proteases (13). This process is hampered in the presence of Vag8, which we expect to bind to C1-INH, and consequently interfere with the protease inhibition allowing for enhanced activation of the contact system (illustrated in Figure 6). As activation of the contact system by bacteria is often induced by membrane-bound components (16), Figure 6 depicts the activation of the contact system on the bacterial surface. However, we cannot exclude that activation occurs both on the surface as well as in fluid phase. Our results indicate that blockage of protease inhibition is essential for *B. pertussis* wild type strain B1917 induced activation of the contact system as in the absence of Vag8, C1-INH is free to inhibit the contact system proteases and, therefore, activation of the contact system is not induced (Figure 3). Whether *B. pertussis* can also interact with the PK inhibitor alpha-2-macroglobulin (45) remains to be investigated.

Here we show for the first time that *B. pertussis* can activate the contact system, which as a bacterium is not unique (15, 36–42). Other bacteria have been shown to activate this system *via* polyphosphates, which are present on *E. coli, Vibrio cholerae, Corynebacterium diphtheria*, and *Haemophilus influenzae* but also on *B. pertussis* (46, 47). Moreover, LPS present on Gram-negative bacteria have been implicated in the activation of the contact system *in vitro* (48, 49). Contact system proteins were furthermore shown to assemble on the bacterial surfaces of *Salmonella typhimurium* and *E. coli* resulting in the release of BK (15). An increase in BK at the site of infection may cause leakage of plasma and be beneficial for the bacteria as this will provide bacteria with nutrients (16). Nonetheless, BK triggers endothelial permeability resulting in infiltration of leukocytes (11). Moreover, cleavage of HK results in the generation of the antimicrobial peptide NAT-26 which could drive bacterial killing (38). Hence, whether activation of the contact system is beneficial or detrimental for the bacteria remains unclear. Contact system protein assembly on *E. coli* occurs on curli pili (42). As we do not observe HK cleavage in the presence of the *B. pertussis* knockout strain B1917ΔVag8, we expect that although the *B. pertussis* membrane-associated polyphosphates or LOS may trigger the contact system, attenuation of C1-INH *via* sequestration by Vag8 is essential for full activation of this system. Alternatively, bacteria can express proteases that actively cleave contact system proteins such as staphopains of *Staphylococcus aureus* or streptokinase of *S. pyogenes*, which both cleave HK releasing BK (38, 50). *B. pertussis* is unique in enhancing the activation of the contact system by producing a protein, Vag8, which inhibits the inhibitory function of the contact system regulator C1-INH.

Infection with *B. pertussis* results in the disease whooping cough, which is typically associated with fits of coughs (or paroxysms) followed by a typical high-pitched whoop. These coughing fits generally persist weeks after the bacterium has been cleared and contribute greatly to the morbidity caused by this disease (51). To date, it is not completely understood what causes this type of cough. The persistence of a chronic cough in the absence of a stimulus is not unique to pertussis but has also been observed in patients on angiotensin converting enzyme inhibitors (ACEi) that are being treated for hypertension (32). ACE, which is produced by lung endothelial cells, breaks down BK (52). The cough associated with ACEi often remains for several days or weeks after the patients have withdrawn from taking the drug. Although the mechanism of ACEi-induced cough remains unresolved, there are indications that BK, of which the levels are increased during ACEi treatment, might be involved (53). The contact system is not only present in plasma but also in the lungs (54) and respiratory administration of BK to guinea pigs but also humans evokes a paroxysmal cough much like the cough associated with pertussis (55). In guinea pigs, this cough was shown to be induced by BK activating the B_2_ receptors on the bronchopulmonary C-fibers (56). These receptors are also expressed in humans on epithelial cells, fibroblasts, and endothelial cells of the bronchial lamina propria (57). It is likely that *B. pertussis* infection results in increased levels of BK in the lungs as we have shown HK cleavage in the presence of *B. pertussis* wild type strain B1917 and BK is a cleavage product of HK. Consequently, we speculate that *B. pertussis*-induced activation of the contact system may be involved in the induction of pertussis-specific cough and thus transmission. Moreover, the activation of the contact system may also play a role in lung pathology. Lung lesions caused by an infection with *S. typhimurium* were shown to be prevented upon inhibition of the contact system in a rat model (40). Fatal *B. pertussis* infection is also characterized by lung lesions (58) and hence the increased BK levels in the lungs following an infection with *B. pertussis* may contribute to lung pathology. Further research needs to be conducted to really understand the role of the activation of the contact system on pertussis pathology.

In light of the re-emergence of pertussis, it has become evident that the development of a novel pertussis vaccine is necessary (5). One of the potential proteins that could be included in such a vaccine is Vag8. Vaccination with Vag8, which was previously only known as a complement evasion molecule of *B. pertussis*, has been shown to give rise to antibodies which protect mice from infection following a *B. pertussis* challenge (59). Moreover, Vag8-specific antibodies have been detected in pertussis patients indicating that Vag8 is produced by *B. pertussis* during human infection (60). Next to the proposed possibility of including Vag8 in a novel acellular pertussis vaccine, this protein is also a component of the OMV-based pertussis vaccine and the live attenuated BPZE1 vaccine that are currently being investigated (61, 62). Vag8 is highly present on OMVs of *B. pertussis* making up 34–50% of the total OMV proteins and can also be found on the bacterial membrane (18, 20, 23). Due to Vag8’s high abundance on OMVs, presence on the outer membrane of *B. pertussis* and the protective effect of this protein as a potential vaccine antigen, the overactivation of the contact system described here, together with the degradation of essential complement protein described earlier (18) may have implications for the inclusion of Vag8 in novel pertussis vaccines. These side effects could include local C1-INH deficiency with consequences for complement and contact system mediated adverse reactions (63). Inhibition of C1-INH could result in increased BK formation which may mediate increased inflammation at the site of vaccination. Moreover, consumption of complement proteins resulting in decreased complement activation following vaccination could have a negative effect on the induction of memory B-cells which are normally induced *via* interactions between C3d-tagged microorganisms or immune-complex antigens and complement receptor 2 (64, 65). It may be advisable to modify Vag8 before the potential inclusion of this antigen in novel pertussis vaccines in order to avoid side effects that could be induced by binding of Vag8 to C1-INH.

In conclusion, we show that Vag8 enhances contact system activity and is mainly responsible for the observed activation of the contact system induced by *B. pertussis*. We propose that this is the result of C1-INH binding by Vag8. This potent C1-INH inhibitor secreted by *B. pertussis* not only mediates complement evasion but also an overlooked interaction between the contact system and the respiratory pathogen *B. pertussis* that may contribute to its pathogenicity and virulence.

## Ethics Statement

The study was conducted using blood donation from healthy adults for plasma collection and according to the principles expressed in the Declaration of Helsinki. Written informed consent was obtained from all blood donors before collection and samples were used anonymously. Approval was obtained from the medical ethics committee of the University Medical Centre Utrecht.

## Author Contributions

EH, SM, AC, SJ, and IJ performed the experiments. EH and SJ drafted the figures. IJ, SM, and CM were involved in the study design. EP and IJ were responsible for funding. EH, SJ, and IJ wrote the manuscript. All authors approved the manuscript’s final version.

## Conflict of Interest Statement

The authors declare that the research was conducted in the absence of any commercial or financial relationships that could be construed as a potential conflict of interest.

## Supplementary Material

Figure S1 Substrate assay kinetics. Substrate conversion, referred to as kallikrein-like activity, was measured in a kinetic fashion up until 30 min. **(A)** 1 µg/mL βFXIIa was incubated alone or with 10 µg/mL complement regulator C1 inhibitor (C1-INH) alone or in combination with different concentrations of virulence-associated gene 8 (Vag8) (2.5, 5, 7.5, 10, 12.5, 15, and 30 µg/mL). **(B)** 0.5 µg/mL plasma kallikrein (PK) was incubated alone or with 10 µg/mL C1-INH alone or in combination with different concentrations of Vag8 (2.5, 5, 7.5, 10, 12.5, 15, and 30 µg/mL). **(C)** 60% plasma was incubated with Vag8 (5, 7.5, 15, and 30 µg/mL) following 10 min pre-incubation with 0.5 µg/mL βFXIIa. Incubation of plasma with βFXIIa alone either without or with pre-incubation served as a control. In the main text, substrate conversion is reported at the 10 min time point. Data represent the mean of three separate experiments.

Figure S2 The negative control BrkA does not interfere with the inhibition of βFXIIa and plasma kallikrein (PK) by complement regulator C1 inhibitor (C1-INH). **(A)** Bordetella resistance to killing A (BrkA) (30 µg/mL) has no effect on the kallikrein-like activity of 1 µg/mL βFXIIa in the presence of 10 µg/mL C1-INH when compared to 1 µg/mL βFXIIa in the presence of 10 µg/mL C1-INH alone. **(B)** BrkA (30 µg/mL) has no effect on the kallikrein-like activity of 0.5 µg/mL PK in the presence of 10 µg/mL C1-INH when compared to 0.5 µg/mL PK in the presence of 10 µg/mL C1-INH alone. Data represent the mean ± SEM of three separate experiments.
