# Supplementary figures and images for: Virulence Associated Gene 8 of Bordetella pertussis Enhances Contact System Activity by Inhibiting the Regulatory Function of Complement Regulator C1 Inhibitor
Source: Front Immunol. 2018 Jun 4;9:1172. doi: 10.3389/fimmu.2018.01172 (PMC5994690; doi:10.3389/fimmu.2018.01172)

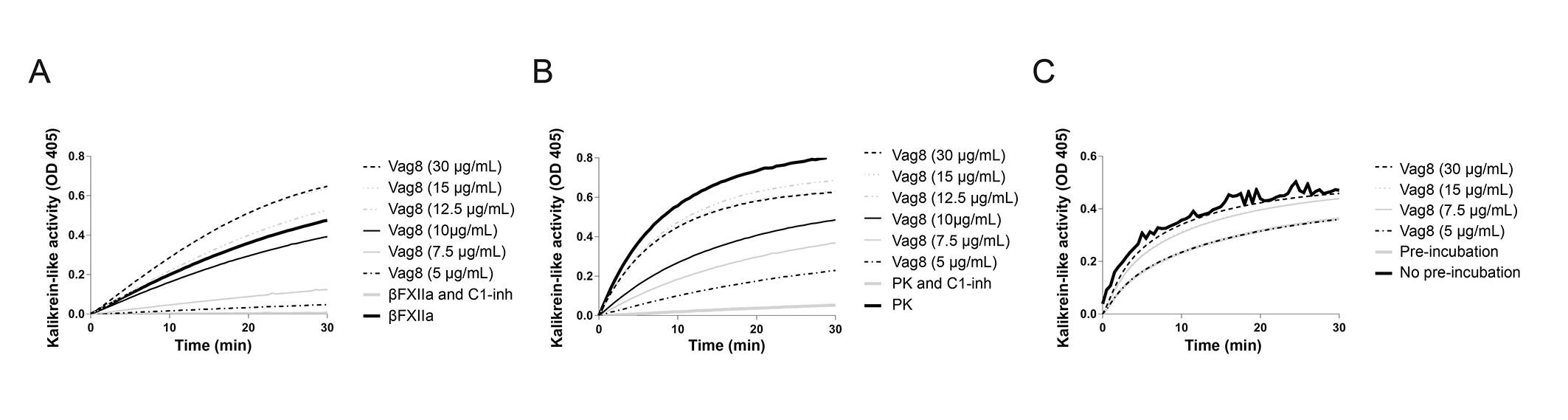

Supplement: Figure S1 — Substrate assay kinetics. Substrate conversion, referred to as kallikrein-like activity, was measured in a kinetic fashion up until 30 min. (A) 1 µg/mL βFXIIa was incubated alone or with 10 µg/mL complement regulator C1 inhibitor (C1-INH) alone or in combination with different concentrations of virulence-associated gene 8 (Vag8) (2.5, 5, 7.5, 10, 12.5, 15, and 30 µg/mL). (B) 0.5 µg/mL plasma kallikrein (PK) was incubated alone or with 10 µg/mL C1-INH alone or in combination with different concentrations of Vag8 (2.5, 5, 7.5, 10, 12.5, 15, and 30 µg/mL). (C) 60% plasma was incubated with Vag8 (5, 7.5, 15, and 30 µg/mL) following 10 min pre-incubation with 0.5 µg/mL βFXIIa. Incubation of plasma with βFXIIa alone either without or with pre-incubation served as a control. In the main text, substrate conversion is reported at the 10 min time point. Data represent the mean of three separate experiments. [file Image_1.TIF]

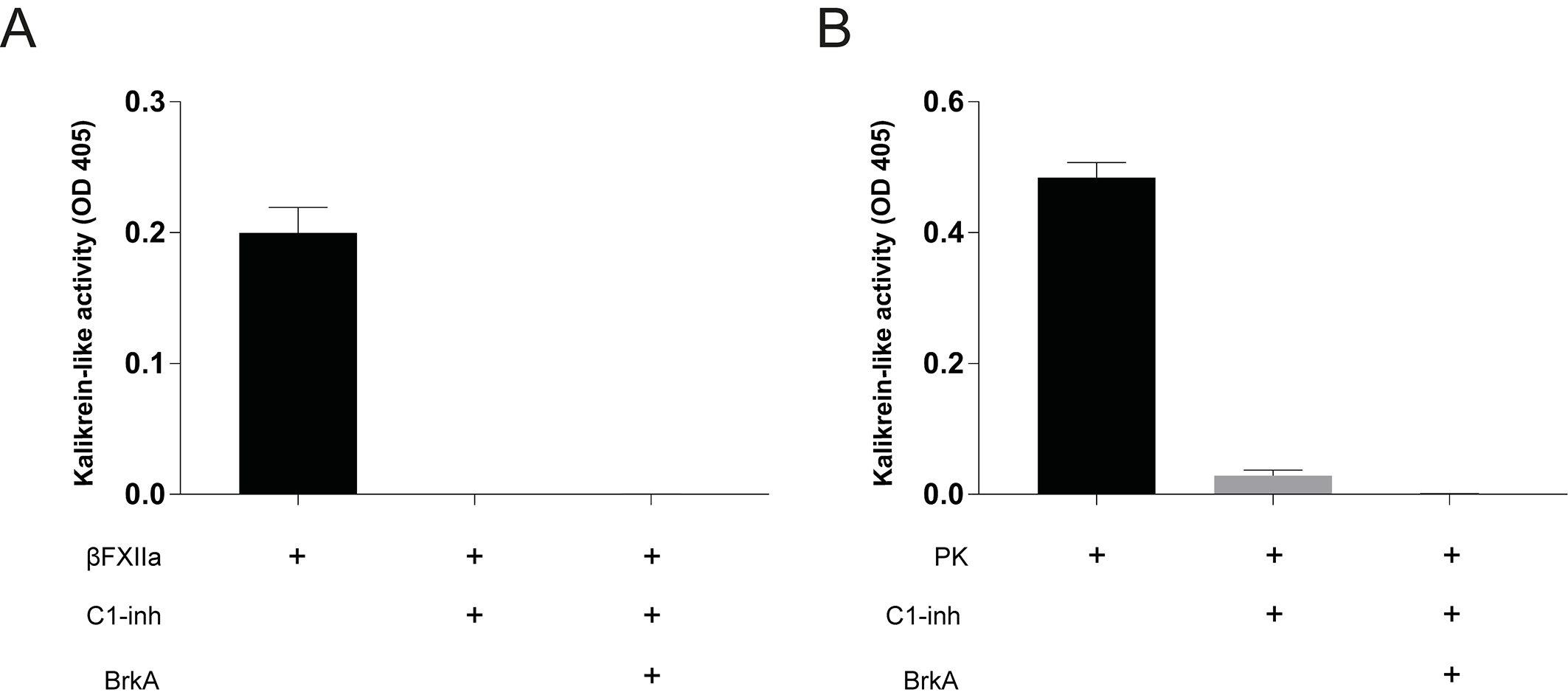

Supplement: Figure S2 — The negative control BrkA does not interfere with the inhibition of βFXIIa and plasma kallikrein (PK) by complement regulator C1 inhibitor (C1-INH). (A) Bordetella resistance to killing A (BrkA) (30 µg/mL) has no effect on the kallikrein-like activity of 1 µg/mL βFXIIa in the presence of 10 µg/mL C1-INH when compared to 1 µg/mL βFXIIa in the presence of 10 µg/mL C1-INH alone. (B) BrkA (30 µg/mL) has no effect on the kallikrein-like activity of 0.5 µg/mL PK in the presence of 10 µg/mL C1-INH when compared to 0.5 µg/mL PK in the presence of 10 µg/mL C1-INH alone. Data represent the mean ± SEM of three separate experiments. [file Image_2.TIF]
